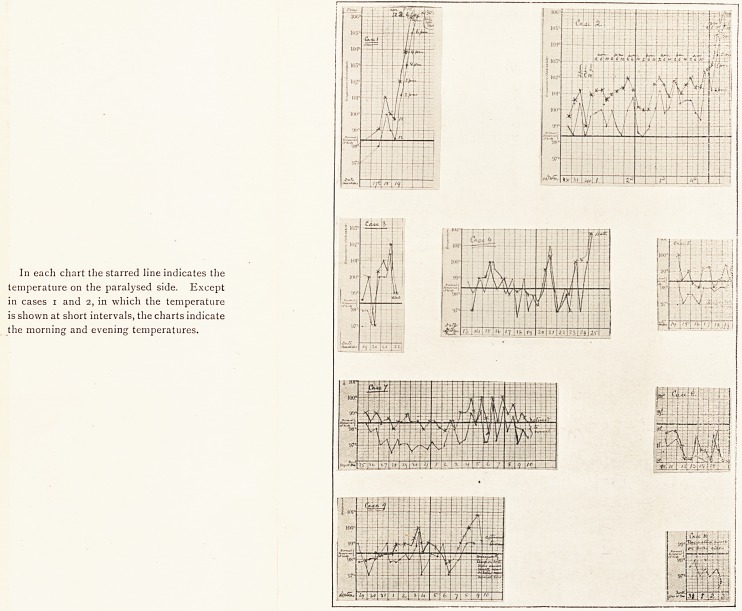# On the Temperature in Cases of Apoplexy, and on the Occurrence (1) of Œdema and (2) of Loss of the Knee-Jerk in the Paralysed Limbs in Hemiplegia

**Published:** 1899-06

**Authors:** J. Michell Clarke

**Affiliations:** Professor of Pathology, University College, Bristol, and Physician to the Bristol General Hospital.


The Bristol

Medico-Chirurgical Journal.

JUNE, 1899.

### ON THE TEMPERATURE IN CASES OF APOPLEXY,

AND ON THE

### OCCURRENCE (1) OF ŒDEMA AND (2) OF LOSS OF THE KNEE-JERK IN THE PARALYSED LIMBS IN HEMIPLEGIA.

BY

J. Michell Clarke, M.A., M.D. Cantab., F.R.C.P.,

*Professor of Pathology, University College, Bristol, and Physician to the Bristol General Hospital.*

The following cases illustrate some of the more interesting points with regard to the temperature of the body in vascular lesions of the cerebral hemispheres. Briefly stated, the chief facts known are—that in cases of cerebral hemorrhage there is an initial fall of the body-temperature; that in rapidly fatal cases this subnormal temperature is maintained, but in others which live for some hours it may be succeeded by a rise to a high level; that in cases which prove fatal after a few days the initial fall is followed by a stationary period of return to the normal or near it, ending in a rise of the temperature before death; and in cases which recover, the temperature after an initial fall and rise returns to normal, or, rather, remains somewhat subnormal for some time. In softening due to thrombosis, there is no initial fall, or it is very slight; the temperature rises, and this is followed by secondary oscillations of considerable amplitude.

Dr. Dana called attention to the fact that in cases of cerebral hemorrhage, accompanied with hemiplegia, the temperature upon the paralysed is higher than that upon the sound side, and that in acute cerebral softening from thrombosis or embolism this difference in temperature is not present. He says: "There are exceptions to this rule which I have laid down, but these exceptions are rare, and are to be explained either on the ground that the hæmorrhage is very small or the acute softening is very extensive." [1]

This difference of temperature on the two sides in hemorrhage may be of practical use in the diagnosis from softening due to thrombosis or embolism. Dr. Dana further says that he has never found any perceptible temperature-disturbance in hemiplegia due to embolism, no matter how severe and pronounced the central disturbance was.

CASE 1.—The first case that I wish to report in illustration of the temperature changes in cerebral hemorrhage is that of a man, aged 48, who had had two attacks of rheumatic fever. There was a history of loss of power in the left side and muffled speech on December 9th, 1898, since which day patient had been confined to bed. On admission, December 17th, patient had left hemiplegia, and was in a dazed, heavy mental state. Early on December 19th he passed into a condition of profound coma, in which he died. The urine contained a considerable quantity of albumin.

The necropsy showed that there was subacute interstitial nephritis, the heart was much dilated, and both ventricular walls were hypertrophied and showed patches of fibroid degeneration. The right cerebral hemisphere was enlarged—the convolutions flattened—and so soft that it was removed with difficulty. The white and grey matter of all the parts of the hemisphere corresponding to the distribution of the middle cerebral artery, with the exception of a narrow zone on the ventral aspects of the optic thalamus and nucleus lenticularis, were yellow, softened, and broken down. A clot was found obstructing this artery, and its branches were thrombosed. A large recent hemorrhage had ploughed up the white substance of the frontal lobe and extended to the surface, about the middle part of the second frontal convolution. The other parts of the brain were normal.

The chart indicates the very high rise of temperature which accompanies the fatal termination in a large cerebral hemorrhage, the temperature on the paralysed side being regularly

[1] *Post-Graduate*, 1896, xi. 316.

a degree higher than that on the sound side, and the interesting fact that thirty minutes after death the temperature on both sides was 106°—that is to say, the temperature on the paralysed side ceased to rise after death, whilst that on the sound side rose one degree. Does this indicate that the healthy side of the brain exerts to some extent an inhibiting influence on the rise of temperature on the opposite side of the body, this inhibition ceasing, of course, with death?

CASE 2 was that of a woman, aged 44, a heavy drinker, who was admitted with right hemiplegia and partial right hemianæsthesia. She was in a very dull, stuporous mental condition. She had had an apoplectic seizure five days before admission.

At the necropsy a large hemorrhage was found to have taken place into the left optic thalamus, and this had later ruptured into the left lateral ventricle, which was filled with bloodclot.

The chart shows the temperature disturbances due to the initial hemorrhage into the thalamus, the temperature on the paralysed varying from half to two degrees higher than that on the sound side; and this difference between the two sides was maintained in the rise of temperature which immediately preceded death, which was due to the effused blood bursting into the lateral ventricle.

CASE 3.—A patient admitted (aged 57) with left hemiplegia and hemianæsthesia, which had come on during the night seven days before admission. He was sensible on admission, but gradually became more dull, and could give no account of himself. After a short period of quiet delirium he gradually became deeply comatose, and so died. Pupils small and contracted. A marked feature in the case was the paralysis of the muscles of the left side of the abdomen, which caused the abdominal wall to be flaccid and bulged out.

*P.M.E.*—The white matter of the right hemisphere was extensively softened and diffluent (white softening). This was most marked in and around the internal capsule, lenticular nucleus, and optic thalamus, extending a little way into the posterior part of the hemisphere and for a considerable distance into the frontal lobe.

The chart shows a rise of temperature before death, with a fall at death; the temperature of the paralysed side was two degrees above that of the sound side on admission, and for the most part slightly above the latter until just before death, when the temperature on each side fell together to the same level. In this extensive softening the temperature difference between the two sides rather resembled that of hemorrhage, but was more irregular.

CASE 4.—A man, aged 46. The symptoms of his illness began with tingling in the left hand and arm, and twitchings of the muscles of this arm and the left leg for a few days. Six days before admission he had a fit, in which he became unconscious, and in which there were twitchings of the muscles of the whole of the left side of the body. This fit was followed by left hemiplegia. After admission he had two or three fits of the same kind, and twitchings of the left arm and calf-muscles were frequently observed. He was dull and drowsy on admission, but subsequently slightly improved; he had very little power of movement of either left arm or leg. Drowsiness was markedly increased on the twelfth day of the illness, and on the fourteenth day deepened into coma, in which he died on the eighteenth day.

A large area of softening was found in the region of the right lenticular nucleus and optic thalamus.

The chart shows that the temperature is irregular, and that on the paralysed and sound side fairly correspond with each other.

CASE 10.—Hemorrhage into the pons Varolii. The chief symptoms were fits, consisting of twitching of the muscles on both sides of the body, with stertor, frothing at the mouth, and cyanosis. Apart from the fits, in the intervals between them convulsive twitchings of the muscles occurred. All four limbs were paralysed, and the motions and urine passed unconsciously. There was drowsiness at first, which passed into coma. There was no oculo-motor paralysis, and the pupils on admission were equal and not contracted. On the last day of life there were irregular, horizontal, oscillating movements of the eyeballs, and alternating contraction and dilatation of the pupils, which were, however, never strongly contracted. The face was slightly drawn to the right; the abdomen was retracted. The superficial reflexes were normal on admission, and there was double ankle-clonus.

*Post mortem* the kidneys were contracted and granular. Two hemorrhages were found in the pons—one on the left side, about $\frac{1}{2}$ inch in long (transverse) diameter, just above upper end of fourth ventricle, in the tegmental region, dorsal to the fillet; the other, about $\frac{1}{8}$ inch in diameter, a little lower down, close to the raphe, at the same level in the pons as the first hemorrhage and on the right side.

The temperature was persistently subnormal and identical on both sides of the body. It is interesting to note the subnormal temperature in the presence of frequent convulsions.

As to the mode of production of a high temperature under such conditions, one must premise that it is not due to muscular convulsions, for these are often absent. Although the subject is an obscure and difficult one, it is hardly to be doubted that the thermo-taxic mechanism is the one at fault. The arguments for the location of the thermo-taxic mechanism in the central nervous system need not be recapitulated; they are well known. This localisation has been further established by numerous experiments to be probably in or near the corpus striatum. Thus Hale White,[1] experimenting on rabbits, found that "lesions of the corpus striatum, if not large enough to cause shock and hemorrhage, lead to a considerable rise of temperature, on the average equal on the two sides of the body, even if only one corpus striatum is damaged." Although lesions of the septum lucidum and posterior part of the upper surface of the cortex in rabbits also caused a rise of temperature, his experiments showed that injuries to the corpus striatum were by far the most important.

The normal regulation of heat is effected by variations in the loss of heat—for a greater amount of heat is produced than is actually necessary—"the heat-producing activities of the organism tend to exceed . . . . its requirements."[2] The heat-regulating mechanism being disordered in cerebral lesions, the temperature tends to rise. The observation that although the temperature rises on both sides of the body, it remains lower on the side of the lesion, is difficult to explain; possibly the sound hemisphere, although much disordered, may still exert some controlling influence on the temperature of the opposite side of the body. In this connection the fact that in the first case the temperature on the sound side rose one degree—to the level of that on the paralysed side—after death is interesting.

In the remaining cases recovery took place, and therefore the diagnosis was not confirmed by *post-mortem* examination; but the cases were selected as being ones in which the diagnosis from clinical symptoms appeared reasonably certain. The first, No. 5, illustrates again the difference between the temperature on the two sides in hemorrhage; No. 7, a similar, but slighter, difference in a case of cerebral syphilis, probably from occlusion of a vessel; No. 8, the subnormal temperature which may persist some weeks in hemiplegia; and No. 9, a want of correspondence between the temperature in the axilla and the surface temperature.

[1] *Brit. M. J.*, 1891, i. 150.

[2] Burdon-Sanderson, "The Doctrine of Fever"; Clifford Allbutt's *System of Medicine*, 1896, vol. i., p. 150.

Case 5.—In this case, although there was no autopsy, the lesion was almost certainly a hemorrhage into the right cerebral hemisphere. The chart shows the difference of temperature on the two sides (paralysed highest) seven days after the apoplectic stroke; after another week the temperature became normal on both sides. Another point of interest was the marked diminution of the knee-jerk in the paralysed leg; it could only be obtained with great difficulty. At the same time rigidity came on in the usual way, and was quite marked one month after the fit. The leg became very tender on movement, and the left knee-joint was swollen and tender.

Case 7.—From a case of hemiplegia due to syphilitic occlusion of a branch of the right middle cerebral artery. The chart shows a difference between the two sides of at first one to two degrees, the temperature on the sound side being markedly subnormal; then follow, as recovery proceeds, a number of rather wide oscillations lasting for about a week, subsequently the temperature on both sides returned to normal.

Case 8.—This chart is taken from a case of hemiplegia seven weeks after the onset. The subnormal temperature on both sides of the body is noticeable at so long a period after the initial lesion; as the patient only came under observation some time after the seizure and recovered, the exact nature of it could not be ascertained. It was probably a hemorrhage. There was no albuminuria.

In some cases of hemiplegia which are associated with disease of the kidneys, a persistent low temperature may be noted, but there was no such reason for it in this case.

Case 9 was one of hemiplegia probably due to softening from thrombosis in the course of chronic Bright's disease. The chart shows that the difference in temperature between the two sides is slight and inconstant. On the 5th August, when the temperature in the axilla was half a degree higher on the paralysed than on the sound side, the surface-temperature was nearly 1½ degrees lower on the former than on the latter. This case presented another symptom sometimes met with in hemiplegia—excessive tenderness to touch or movement of the paralysed limbs. The patient screamed loudly if they were moved or touched. This was not attributable to rigidity of the limbs, as the rigidity was only slight. Another symptom she suffered from was well-marked œdema of the paralysed side of the body only, especially of the arm and leg. Associated with this appeared trophic disturbances in the shape of a sore on the heel and a blister if the skin was thrown into a crease or fold anywhere by her position in bed.

The presence of this unilateral œdema is, I think, to be attributed to a change in the innervation or nutrition of the vessel-walls, and may be compared to the experimental œdema in the leg produced after division of one sciatic nerve in the leg after the production of artificial hydræmia.

When albuminuria is present the above seems to be the most reasonable explanation of œdema of the paralysed side, and is in accordance with known factors in the production of œdema. But in other cases the etiology is not to be thus

In each chart the starred line indicates the temperature on the paralysed side. Except in cases 1 and 2, in which the temperature is shown at short intervals, the charts indicate the morning and evening temperatures.

explained, for a similar œdema may occur in hemiplegia in cases in which albuminuria is absent, and there is no reason to suspect disease of the kidneys. Thus in a woman who suffered from mitral disease, and in whom left hemiplegia was presumably due to thrombosis followed by softening, but who had no albuminuria nor other evidence of kidney-disease, five weeks after the attack œdema of the paralysed arm and hand came on, and the limb became painful to the touch, and there was at the same time marked rigidity. Indications of vaso-motor disturbance were found in cold sweats and lividity confined to the hand and forearm, and this limb was always colder than the right. The leg was less affected, and began to recover power early. There was, of course, no evidence of local pressure upon or of disease of the vessels of the left arm.

In both these cases the appearance of œdema was accompanied by pain, which was more or less constant, but much increased by movement. This pain was not the same as that which is aroused by forcibly moving the parts of a rigidly-contracted limb, and was apparently not connected with the rigidity present. The appearance of this painful œdema is an unfavourable sign in the prognosis as to recovery of power in the limbs, and is difficult to relieve by treatment. The first of the above two cases (case No. 9) exhibited also another phenomenon which I have observed in three cases of hemiplegia—a marked diminution or sometimes loss of the knee-jerk in the paralysed leg, the exact contrary of what usually obtains. I append brief notices of the two other cases.

A man, aged 71, was admitted three days after onset of a seizure, probably due to thrombosis, which occurred in the night, and was accompanied by twitchings of the right side of the face, right arm and leg, but not by complete loss of consciousness. Right hemiplegia resulted, and was at first accompanied by anæsthesia of the whole of the right lower extremity, which was soon reduced to a patch of anæsthesia in the area of distribution of the N. cutaneus femoris. There was slight concentric contraction of the right visual field and a moderate amount of albumin in the urine. He recovered power gradually, and was able to walk out of the Hospital four weeks later. The right knee-jerk was completely absent, and could never be obtained by any method or by reinforcement, whilst the left knee-jerk was active and the superficial reflexes normal. The leg became moderately stiff, but was never very rigid; it was not, however, flaccid. Subsequent examinations showed the right knee-jerk to be always absent.

In the case of a man, aged 65, with left hemiplegia, partial, not complete, hemianæsthesia, left hemianopsia, and with slight movements of athetosis in the left hand, the left knee-jerk was also absent. On the right side it was normal. The other reflexes were normal, and the amount of rigidity present in the paralysed limbs was only moderate.

I cannot give any explanation of the loss of the knee-jerk on the side of the hemiplegia; especial care was taken to ascertain that it was really absent. In two of the cases there was also some anæsthesia on the affected side; but in the other the sensory disturbance was in the opposite direction, consisting of increased pain and tenderness.

## Figures and Tables

**Figure f1:**